# On the ability of standard and brain-constrained deep neural networks to support cognitive superposition: a position paper

**DOI:** 10.1007/s11571-023-10061-1

**Published:** 2024-02-04

**Authors:** Max Garagnani

**Affiliations:** 1 Department of Computing, Goldsmiths – University of London, London, UK; 2 Brain Language Laboratory, Department of Philosophy and Humanities, Freie Universität Berlin, Berlin, Germany

**Keywords:** Concept combination, Multi-item working memory, Brain-constrained modelling, Semantic representations, Artificial cognitive system, Cell assembly, General intelligence

## Abstract

The ability to coactivate (or “superpose”) multiple conceptual representations is a fundamental function that we constantly rely upon; this is crucial in complex cognitive tasks requiring multi-item working memory, such as mental arithmetic, abstract reasoning, and language comprehension. As such, an artificial system aspiring to implement any of these aspects of general intelligence should be able to support this operation. I argue here that standard, feed-forward deep neural networks (DNNs) are unable to implement this function, whereas an alternative, fully brain-constrained class of neural architectures spontaneously exhibits it. On the basis of novel simulations, this proof-of-concept article shows that deep, brain-like networks trained with biologically realistic Hebbian learning mechanisms display the spontaneous emergence of internal circuits (cell assemblies) having features that make them natural candidates for supporting superposition. Building on previous computational modelling results, I also argue that, and offer an explanation as to why, in contrast, modern DNNs trained with gradient descent are generally unable to co-activate their internal representations. While deep brain-constrained neural architectures spontaneously develop the ability to support superposition as a result of (1) neurophysiologically accurate learning and (2) cortically realistic between-area connections, backpropagation-trained DNNs appear to be unsuited to implement this basic cognitive operation, arguably necessary for abstract thinking and general intelligence. The implications of this observation are briefly discussed in the larger context of existing and future artificial intelligence systems and neuro-realistic computational models.

## Introduction

### Premise

The capacity of an (artificial or natural) cognitive system to recall and maintain *simultaneously active* in its working memory two or more internal representations is known as “superposition” in neurocomputational modelling (Greff et al. 2020; Milner 1974; Rosenblatt 1962; von der Malsburg 1986), “concept combination” in psychology and philosophy of mind (Costello and Keane 2001; Hampton 1991, 1997; Rips 1995; Wisniewski 1997), and “multi-item working memory” in cognitive neuroscience (Axmacher et al. 2010; Jensen and Lisman 2005; Lara and Wallis 2014; Yakovlev et al. 2005). This cognitive ability allows us to mentally combine instances of any two (or more) conceptual categories stored in semantic memory (Tulving and Madigan 1970). For example, having previously acquired the concepts of “apple” and “car”, one can conjure up a mental image combining (in any arbitrary spatial arrangement) two instances of these concepts. Crucially, this is possible even when the semantic categories were learned *independently*, i.e., no two samples of such concepts were ever “experienced together” (in the example, assume the cognitive agent has never seen an apple and a car in the same scene). Indeed, the ability to combine familiar items’ representations in novel, arbitrary ways may well be the mechanism underlying the human capacity to develop *new* internal representations such as abstract concepts, which likely build upon yet go well beyond what is normally perceived in the environment (Barsalou and Wiemer-Hastings 2005; Borghi and Mazzuca 2023; Pulvermüller 2013).

The present article focusses on the ability of a system to dynamically (i.e., temporarily) co-activate the representations of two previously acquired concepts while still maintaining such internal representations distinct and functionally separate (the latter aspect is elaborated on further below). It does not deal with the second important issue mentioned above, namely, the ability to use the result of a superposition operation to construct a novel conceptual item. This choice is motivated by the fact that the presence of a mechanism supporting the former process must be a prerequisite for the implementation of the latter. In other words, the emergence of a new internal representation combining previously existing ones requires a system to be able *at least* to support the co-activation of such multiple instances (it is difficult to see how a system unable to superpose its previously acquired representations could develop new ones that encode such states of co-activation). Thus, it seems justified to start by addressing the more basic and fundamental issue of items superposition itself, leaving the latter topic for a separate, dedicated treatment.

The idea of superposition is closely linked to the concept of working memory (WM), which can be defined as the brain/cognitive system that enables temporary storage and manipulation of information needed for advanced tasks such as language comprehension, problem solving, and abstract reasoning. Intuitively, WM can be thought of as a “mental workspace” where information (items, concepts, goals) relevant to the task at hand is retained for a short time and actively worked on (Baddeley 2003; Eriksson et al. 2015; Fuster 1999; Goldman-Rakic 1995; Miller et al. 2018). Importantly, WM has a limited capacity: the average person can maintain co-active only up to four or five items (Cowan 2001; Cowan et al. 2007); this capacity limitation significantly influences higher cognitive functions like reading, fluid reasoning, and general intelligence (Conway et al. 2003; Engle et al. 1999; Lara and Wallis 2014). A large and growing body of works investigating computational modelling of WM function and memory cells’ emergence in the cortex exists (e.g., Amit and Brunel 1997; Camperi and Wang 1998; Compte et al. 2000; Deco and Rolls 2003; Mongillo et al. 2008; Pulvermüller and Garagnani 2014; Tagamets and Horwitz 2000; Zipser et al. 1993, to name a few). The focus of this brief article is not to propose a novel idea or candidate set of neural mechanisms, but to contrast two existing types of neurocomputational architectures—deep, brain-constrained and “standard” feed-forward multilayer neural networks—in terms of their ability to support a specific aspect of WM, namely, the simultaneous activation (temporary storage) of multiple (two or more) items. While some neurobiologically constrained models that spontaneously exhibit this ability do exist (Szatmáry and Izhikevich 2010; Ursino et al. 2023), these either simulate just a single area, or implement *ad hoc*, neuroanatomically unrealistic between-layer connections. As argued later, using a deep (i.e., multi-area) hierarchy with connectivity closely mimicking features of real cortico-cortical projections may be crucial for the emergence of internal circuits suitable to support cognitive superposition.

It is important to clarify here the need for the above-mentioned property of the superposition function—namely, that the cognitive system must be capable to co-activate two (or possibly more) internal representations *while still maintaining these representations distinct*. This constraint is needed to ensure that the system does not fall prey to the well-known binding problem (Milner 1974), often referred to as the “superposition catastrophe” (Page 2000; Rosenblatt 1962; von der Malsburg 1986), introduced below.

**Fig. 1:**
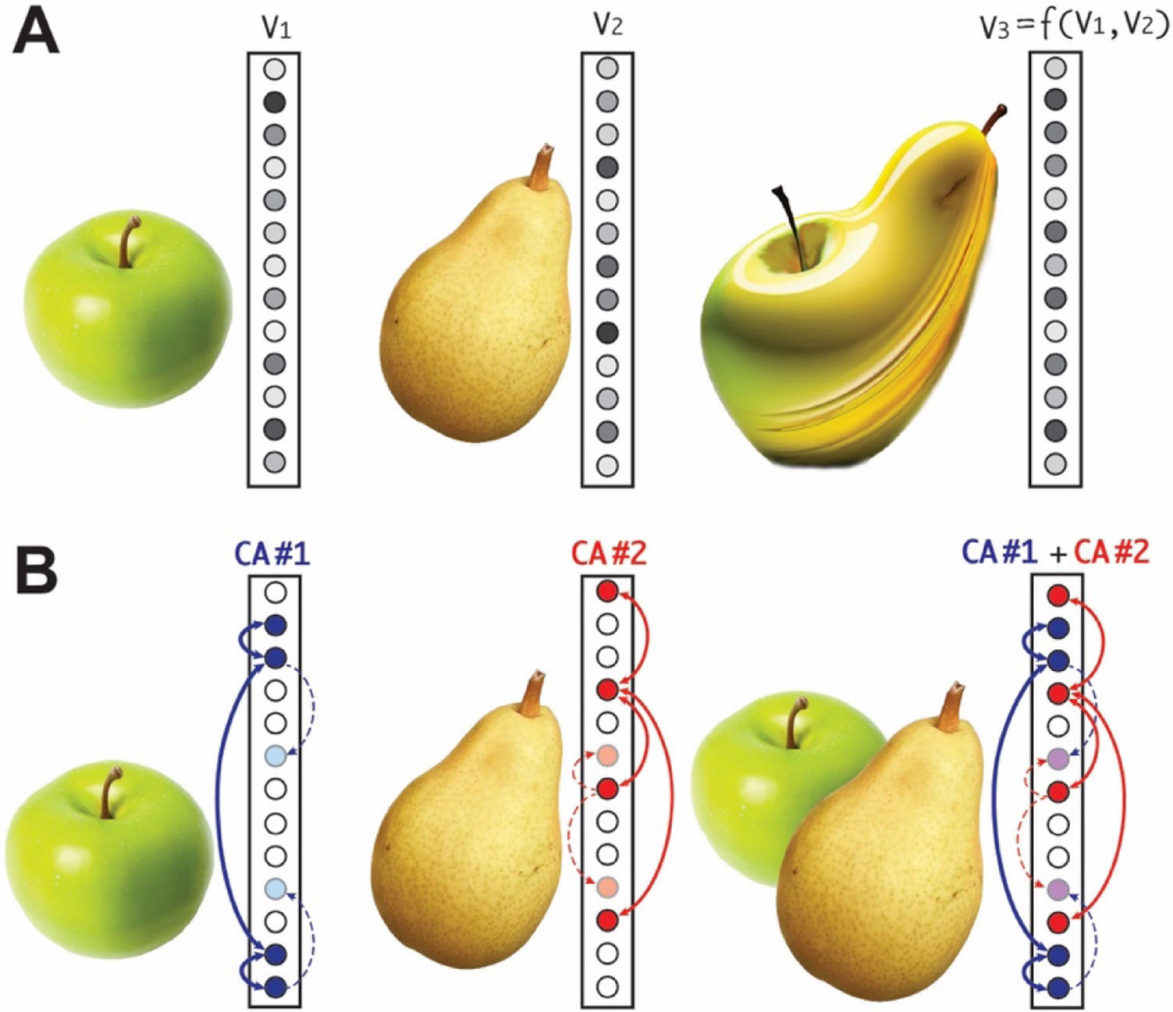
Superposition in standard (**A**) and brain-constrained (**B**) neural networks. The vertical arrays represent a neural network’s set of nodes, whose activity levels are indicated by grey scales and colour shadings. **A:** In standard feed-forward NNs trained with back-propagation, distinct sensory (or conceptual) items are learned as distinct vectors of *graded* activities over the *same set of nodes* (which here may be coding for visual features of objects, such as colour, shape, etc.). As any two such activity vectors are generally not orthogonal, their sum (co-activation) leads to a new vector from which the original components cannot be uniquely identified. In the example, superposing the learned representations for ‘green apple’ (V_1_) and ‘yellow pear’ (V_2_) produces an ambiguous vector V_3_ (depicted as a “blend” of the two original items) which could also be the result of co-activating a ‘yellow apple’ and a ‘green pear’, or an infinite number of other pairs of items having some in-between colours and shapes. **B:** In the class of brain-constrained networks in focus here (trained with a biologically constrained Hebbian learning rule – see main text), the network correlates of distinct input items spontaneously emerge as distinct cell assembly (CA) circuits made up of *mostly disjoint* sets of strongly linked cells, each CA behaving as a functionally distinct unit having two activity states (“on” and “off”). In the example, activating the learned representation for a ‘green apple’ involves the full “ignition” of CA#1, depicted as a set of blue circles (active nodes) and arrows (links through which activity is reverberating). Similarly for the circuit (cor)responding to a ‘yellow pear’ (CA#2, red circles & arrows). As the most active cells of the two CAs – known as the CAs' kernels (Braitenberg 1978) – do not overlap, the network's activity states respectively induced by the ignition of CA#1 and CA#2 are *quasi* *orthogonal* (i.e., the strongly “on” nodes of one state are “off” in the other, and vice versa). Although two CAs may share a small portion of their constituent cells (light-blue and light-red circles), these are only weakly linked to the CA kernel (dashed arrows) and do not significantly contribute to its activity. Superposition thus leads to a network state (CA#1 + CA#2) in which both circuits are “on” but remain functionally distinct

In what follows, it is assumed that a cognitive system encodes all items (or concepts) as patterns of activity (vectors) over a set of processing units, the internal (“hidden”) nodes of a network (refer to Fig. 1). This is the representation adopted by modern, deep (i.e., multi-layer) Neural Networks (NNs) (Krizhevsky et al. 2012; LeCun et al. 2015) and, more in general, by all architectures adopting a Parallel Distributed Processing approach (McClelland et al. 1986). In standard (deep) NNs, an “internal representation” can be defined as a state of nodes’ activities mapping an input-layer pattern to a corresponding output-layer pattern, a mapping typically acquired as a result of (gradient-descent) learning. Superposing two such internal representations (i.e., co-activating the respective input patterns) leads to a novel output that *combines* elements of the original ones. This is illustrated in Fig. 1A as a novel object exhibiting morphed features of the two co-activated items (rightmost panel). Indeed, images very much like the one depicted in Fig. 1A (rightmost panel) can be easily generated using a class of NNs known as “Generative Adversarial Nets” (GANs) (Arjovsky et al. 2017; Brock et al. 2018; Goodfellow et al. 2014; Mirza and Osindero 2014; Radford et al. 2015). While the ability of such systems to classify and create realistic images, or recognise speech, rivals our own (e.g., see Baraheem et al. 2023; Smit et al. 2021; Wang et al. 2020 for reviews), one shortcoming of the “fully” distributed code that modern NNs adopt is that the superposition of two learned representations produces a new one which contains elements of both, but in which the original elements can no longer be uniquely identified. In other words, the result of co-activating two distinct input patterns leads to a new activity vector which *does not enable retrieving the exact features of the two components* (Page 2000; Pulvermüller 2023; Vanegdom et al. 2022). This is because, in such neural architectures, distinct entities are generally encoded as *non-orthogonal* vectors over the same set of processing units (see Fig. 1A). In fact, in an *n*-dimensional space, for any given vector V_*z*_ there is an *infinite number *of pairs of non-orthogonal vectors V_*x*_ and V_*y*_ such that V_*x*_ + V_*y*_ = V_z_; thus, the superposition of two (non-null) vectors V_1_ and V_2_ produces a new vector V_3_ which is "ambiguous", in the sense that it could be the result of many different additions of non-orthogonal vector pairs.

In the example shown in Fig. 1A, the components of network state (vector) V_3_ do not allow determining the components of V_1_ and V_2_, even when V_3_ is simply the sum of V_1_ and V_2_ (let alone in the more general case, when V_3_ is a non-linear function *f*() of V_1_ and V_2_). Thus, the characteristic features of the two original objects can no longer be retrieved. An artificial or natural cognitive system must be able to avoid such a “superposition catastrophe”: activating several items in WM should not imply a loss of information about the original entities; rather, the system should be able to *integrate* multiple representations while still maintaining the individual elements distinct, as depicted in Fig. 1B (rightmost panel).

In view of the above considerations, I suggest that a cognitive system may be deemed able to support cognitive superposition if, and only if, both of the following conditions hold:

A. The system is capable to maintain simultaneously active internal representations of any two (or more) arbitrarily chosen, previously acquired sensory (or conceptual) items, as distinct elements;
B. The arbitrarily chosen elements may be such that the system has never “experienced” them together in the past.

Condition B. requires a system to be able to combine representations which were acquired independently of each other and which may have never been co-activated before. In fact, a system that must have been exposed a priori to the simultaneous presence in the environment of each possible combination of items it may need to reason about would be very limited: as argued below, the ability to combine internal representations of previously learned, familiar objects in a new, not previously experienced way seems to be a pre-requisite for abstract reasoning, language, and creative thinking, faculties that characterise our species and are key to general intelligence (Arbib and Bonaiuto 2016; Gazzaniga et al. 2018).

In the remainder of this short article I argue that (1) superposition is a crucial building block for the emergence of advanced thinking skills that any artificial cognitive system should aim to support; (2) standard deep NNs are trained in a way that makes their internal representations inadequate to implement superposition; and (3) a class of deep, brain-like neurocomputational architectures trained with biologically realistic Hebbian-like learning mechanisms exhibit the spontaneous emergence of distributed yet functionally distinct internal circuits having features that enable them to naturally support this fundamental cognitive function.

### Superposition is a fundamental cognitive ability

Superposition operations as defined in the above section potentially underlie our mental capacity to create associations between multiple, previously and independently acquired concepts. This appears to be a key functional feature of our cognitive apparatus.

Anecdotal evidence showing that the human brain supports superposition is provided by a number of direct observations. First, one must be able to activate two object (or conceptual) representations during the same mental operation when, for instance, one wishes to keep in mind both for direct comparison (von der Malsburg 1999). Second, higher-level cognitive functions such as problem solving, mental arithmetic, spatial and abstract reasoning, planning and complex decision making, often considered characteristics of general intelligence, appear to rely heavily on the WM’s ability to store and manipulate several items at the same time (Conway et al. 2003; Engle et al. 1999; Lara and Wallis 2014). Third, language usage constantly requires superposition (Thornton 2021): sentences can contain any arbitrary combination of two (or more) words referring to concepts or objects which may have never been encountered together in the same (physical or conceptual) context before. Given our ability to understand such sentences, it follows that our brain must be able to co-activate the representations of the multiple referent objects a sentence may talk about. Returning to the earlier example, the fact that the sentence containing the words “apple” and “car” in the previous section can be easily understood is direct proof of one’s ability to co-activate the representations of these two concepts in WM. In fact, over the last couple of decades several researchers in the field of neurocomputational modelling of language processing have been proposing the formation and sequentially ordered *co-activation* of cell assembly (CA) circuits—long-term memory traces hypothesized to emerge spontaneously in the cortex as a result of associative mechanisms (Abeles 1991; Braitenberg 1978; Hebb 1949; Palm 1981; Singer et al. 1997; von der Malsburg 1986)—as one of the main mechanisms underlying word learning and the acquisition of syntax and grammar in the brain (Knoblauch and Pulvermüller 2005; Pulvermüller 1999, 2000, 2003a, 2003b, 2013; Pulvermüller and Fadiga 2010; Wennekers et al. 2006).

Lastly, besides its manifest importance in language processing and abstract reasoning and, recently, evidence of it being implicated also in social cognition (Noguchi et al. 2022), superposition—or, rather, addressing the problem of the superposition catastrophe—has been long since associated with modelling and explaining the brain mechanisms underlying visual object perception and recognition (Milner 1974; Rosenblatt 1962; von der Malsburg 1986), as reviewed below.

### Superposition catastrophe in standard neural networks

Albeit designed with engineering goals in mind and not to mimic brain function, modern, Deep and Convolutional Neural Networks (D/CNNs) have been found to exhibit features that reflect properties of some parts of the human neocortex, suggesting common underlying organizational and/or functional principles (Kriegeskorte 2015; LeCun et al. 2015). In fact, when trained to classify a set of stimuli (e.g., images of objects, speech sounds), the hidden layers of DNNs develop types of responses that are, to an extent, similar to those observed experimentally in corresponding hierarchies of cortical areas responsible for processing such stimuli (Kriegeskorte 2015; Richards et al. 2019; Yamins and DiCarlo 2016). For example, DCNNs trained with the gradient-descent rule (or backpropagation) (McClelland et al. 1986; Rumelhart et al. 1986) to classify images of objects or letters were found to be able to explain and predict neural responses observed in corresponding brain areas located in the inferior aspect of the temporal lobes (Güçlü and van Gerven 2017; Khaligh-Razavi and Kriegeskorte 2014; Testolin et al. 2017), part of the so-called “ventral stream” of visual information processing (Mishkin et al. 1983; Ungerleider and Haxby 1994).

The analogy “DNN ≅  hierarchy of areas for sensory information processing”, however, has been put under scrutiny: recent results suggest that DCNNs cannot fully capture higher-level visual representations of real or artificial objects (Gale et al. 2020; Xu and Vaziri-Pashkam 2021a, 2021b); more generally, modern DNNs have been reported to be fragile (Jozwik et al. 2017), exhibit limited generalisation abilities (Greff et al. 2020) and fail to incorporate elements considered essential to attain human-like intelligence (Bishop 2021; Lake et al. 2017; Marcus 2018); cognitive superposition appears to be one of such crucial elements.

Historically, a number of authors recognized that achieving superposition in “standard”—i.e., multi-layer perceptron, gradient-descent trained—neural networks is problematic: DNNs have been claimed to inherently suffer from the already mentioned superposition catastrophe (Milner 1974; Page 2000; Rosenblatt 1962; von der Malsburg 1986). In a NN modelling context, this issue can be formulated as follows: given a network trained to associate input and output patterns (pairs of activity vectors), simultaneously activating two (or more) of the learnt vectors in the input layer leads to a “blended” activation pattern in the output layer which is ambiguous, i.e., which might have been produced by more than just one combination of inputs (see Fig. 1A).

In an attempt to understand why brain-inspired systems such as artificial neural networks turn out to be unable to carry out a fundamental cognitive function the brain effortlessly supports, some authors investigated whether the superposition catastrophe pervasively afflicts all NNs or whether, under certain circumstances, this problem may be overcome. For example, using backpropagation-through-time (Mozer 1995; Werbos 1988), Bowers and colleagues trained a three-layer recurrent network to associate single and superposed input patterns with corresponding (localist) output patterns (Bowers et al. 2014). Their results showed that the network was not only able to learn such associations, but that, as a result of training, many of its hidden nodes spontaneously acquired a high degree of selectivity. The fact that a network explicitly trained to produce the desired superposed output for a set of superposed inputs spontaneously develops so-called ‘localist’ representations in its hidden layers led the authors to conclude that such representations must play a role in the brain (Bowers et al. 2014). However, these results were not replicated: recently, Nicolas Martin showed that an analogous recurrent NN could be trained to produce the correct output for any set of superposed input patterns without giving rise to the emergence of highly selective nodes (Martin 2021). Crucially, both studies fail to demonstrate a network’s ability to superpose two independently learned representations, as required by condition B. in the “Premise” section. In fact, the requirement specified there is that superposition should not need the corresponding items to have been a priori “experienced” together by the system, whereas both Martin and Bowers et al. used networks in which the relevant representations were coactivated in the hidden layer *during training* (Bowers et al. 2014; Martin 2021)*.*

Taking a different approach, a number of scholars (e.g., Burwick 2006; Engel et al. 1991a, b; Hummel and Biederman 1992; Schillen and König 1994; Shastri and Ajjanagadde 1993; Singer 1995) suggested that the brain may solve the superposition catastrophe (and the closely related “binding problem”) by means of rhythmic activity: if all cells encoding the features of the same sensory item (e.g., its colour, shape, size, etc.) fire in synchrony and repeatedly, in a select phase of an oscillatory cycle, then multiple objects can be superposed without any risk of ambiguity, assuming distinct items are allocated distinct phases (cf. Shadlen and Movshon 1999 for a critical review). However, what the vast majority of such studies don’t address is the exact neural mechanisms via which the brain might maintain such precisely timed synchronisation between “distant”, not directly linked neurons, and for long periods of time (several seconds), without suffering from cross-talk and interference, as required to perform cognitive superposition.

## Deep brain-constrained Hebbian-learning nets support Cognitive Superposition

In the above introductory sections I argued that superposition is a fundamental cognitive skill, and reviewed some studies which investigated the ability of backpropagation-trained NNs to support this function. In this section I use results from computational simulations as a proof of concept to show that a class of deep (i.e., multi-area/multi-layer), brain-constrained networks (Garagnani et al. 2016; Garagnani and Pulvermüller 2011, 2016; Garagnani et al. 2008, 2009a, b; Henningsen-Schomers et al. 2023; Pulvermüller and Garagnani 2014; Pulvermüller et al. 2021; Schomers et al. 2017; Tomasello et al. 2017, 2018, 2019), in which distinct, stimulus-specific cell assembly (CA) circuits (Braitenberg 1978; Hebb 1949; Palm 1981) spontaneously emerge as model correlates of input patterns, can support superposition without suffering from interference.

The requirements A and B stated in the Premise as necessary and sufficient conditions for a cognitive system to be able to support superposition can be rewritten, in neural network terms, as follows:

### Definition

*A neural network model is said to support cognitive superposition if, and only if*:

1. It allows co-ativation of any two vectors of hidden nodes’ activities, associated with distinct input items that were never presented together during the training phase; and
2. During co-activation, the two activity vectors are combined in such a way that information about the identity and features of the original components is preserved.

**Fig. 2:**
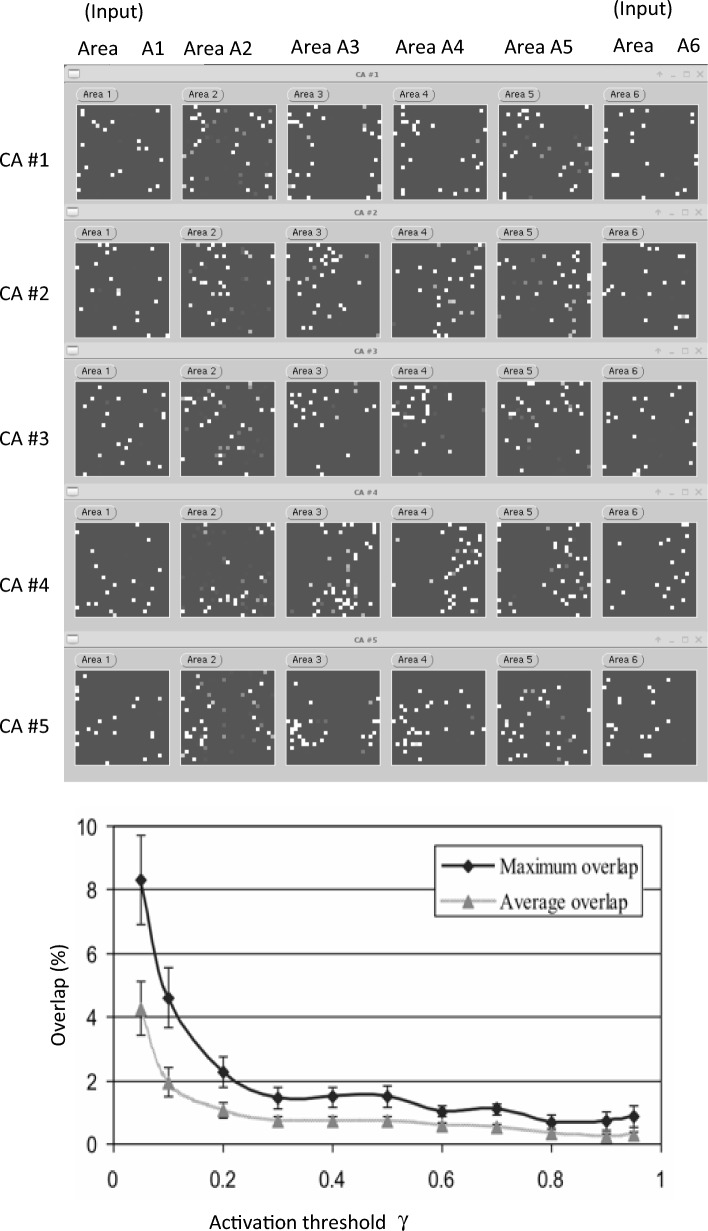
Examples of Cell Assembly (CA) circuits and their overlaps. Top: Five (out of 12 learned) CA circuits emerging in the six-layer deep brain-constrained network used for the present study, having structure, connectivity and learning mechanisms identical to that in (Garagnani et al. 2008). Each network layer (or “area”), depicted as a darker square, consists of 25 × 25 excitatory and 25 × 25 inhibitory (not shown) graded-response cells. Pixels’ brightness indicates cells’ activity levels. Training was implemented by repeated concomitant presentation of (binary) patterns to areas A1 and A6, each pattern activating 19 of the 625 cells. After 3,000 presentations, model areas A2–A5 exhibit distributed sets of cells strongly and selectively responding to each of the input pattern pairs; these cells form the emerging CA circuits. Note that the network response includes also less active cells, which form part of the CA’s “halo” (Braitenberg 1978): these cells are only weakly (and not reciprocally) linked to the strongly active CA cells, the CA’s kernel (see also Fig. 1B). The six areas are serially (next-neighbour) and recurrently linked (not depicted) via sparse, random and topographic projections (see Garagnani et al. 2008 for details). Bottom: mean and maximal overlap (% of shared cells) between the emerging CA circuits are plotted as a function of the threshold γ used to identify them: more precisely, a cell is considered part of a CA circuit if its activity during input stimulation (Top panel) reaches a given level, proportional to γ. Note that the maximal overlap between any pair of CA circuits remains below 5% for a wide range of threshold values (adapted from Garagnani et al. 2008, their Fig. 8)

Figures 2 depicts results of computational simulations obtained with a six-area (or six-layer) deep brain-constrained network analogous to that used in (Garagnani et al. 2008). The top panel (areas A2–A5) illustrates the structure of five representative CAs which emerged as a result of neurobiologically realistic learning. The bottom panel (showing results from simulations obtained with an analogous architecture) plots percentage overlap between pairs of learnt CAs as a function of the threshold γ, which was used to determine the set of cells forming a CA circuit: more specifically, a cell was “counted” as belonging to a given CA if, and only if, its graded response during stimulation with the relevant input patterns reached level γ ⋅ *M*, where *M* was the output of the maximally responsive cell for that pattern (for details, see Garagnani et al. 2008; Garagnani et al. 2009a, b).

**Fig. 3:**
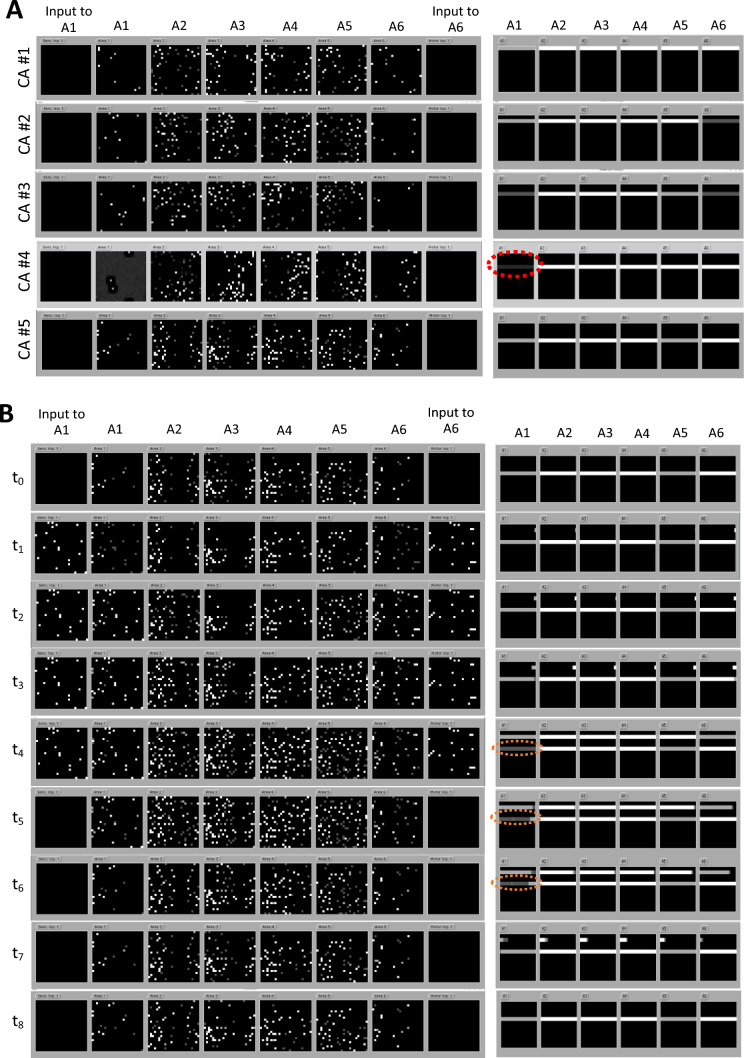
Cell assemblies in deep brain-constrained neural networks, and their superposition. **A**: Snapshots of current and recent network activity during self-sustained reverberation of each of the five cell-assembly circuits shown in Fig. 2. (Left): Each of the five rows depics a snapshot of the network activity (including the two input areas) taken when one of the five CAs circuits showin in Fig. 2-Top was fully active (“ignited”) and exhibited reverberant activity in absence of any input. The activity within the circuit was self-sustained, and the system was in a fixed-point attractor state (though minor oscillations around the fixed point were observed). Also note that only a subset of the cells identified in Fig. 2-Top as forming the CA circuits is showing high activity levels; in particular, the most “peripheral” areas A1 and A6 (where the input patterns were presented during training) contain only a few active CA cells, suggesting that the kernel of the CA circuits lies mainly in the four “central” areas (A2-A5) of the arhictecture. The reason for the only partial binding (and reconstruction) of the stimulus patterns into the cell assembly is to be found in the sparse – as opposed to ‘*all-to-all*’ – between- and within-area connectivity of the network: due to the low density of recurrent and between-area projections, some of the cells directly stimulated by the inputs to areas A1 and A6 happen to be linked neither to other co-active cells in such areas nor to (CA) cells the patterns indirectly activate in other layers. (Right): Each of the five snapshots shows the recent history of the total within-CA activity (calculated as the sum of the responses of all cells belonging to a CA circuit) for the twelve CAs the network had learned. Specifically, each of the six smaller quadrants displays the raster plots of the within-CA activities during the last 150 simulation time steps. Within a quadrant, each row shows (using a suitably normalised gray scale) the total activity within each CA circuit (first row for CA #1, second row for CA #2, etc.). Thus, for example, a vertical segment at a given time point reveals that a greater-than-zero portion of CA cells was active in that area. Significant persistent per-area activities within any of the 12 CA circuits are thus visible as bright “bands” on the relevant rows (as CAs #6 – #12 are not depicted, only the first five rows show activity). Note that, consistent with the previous observation of the CA kernels being mostly in the four central model areas, self-sustained CA activities tend to be weaker in the two peripheral areas (A1, A6) – see, e.g., the low percentage of the input pattern reconstructed by reactivation of CA #4 in area A1 (only 2–3 cells out of the original 19-cell pattern), as indicated by the almost invisible gray band in the corresponding quadrant (see red dashed oval). **B**: Representative example of CA superposition. Overall network activity is plotted at nine ordered time points (arbitrarily chosen) during an episode of two CA-circuits’ dynamic superposition. (Left): snapshots of current network activity (six areas and two input patterns). (Right): raster plots of total within-CA activities for the corresponding network states shown on the left. Initially (time t_0_) the network is in a stable state, showing persistent, self-sustained activation of CA #5 (note the bright bands in the fifth row, right-hand side panel). At time t_1_ the inputs to A1 and A6 are set to the patterns that led to the emergence of circuit CA #2 (cf. Figure 2-Top). During the following time steps (t_2_-t_3_), the second CA circuit (CA #2) ignites, with its cells ‘lighting up’ first in A1 and A6 and then rapidly extending to  the central areas. By time t_4_, activity is stable and shows superposition of CAs #2 and #5 (note the corresponding activity bands on rows 2 and 5 in all network areas – Right). In this example, the strengths of the internal links of the second assembly were insufficient to allow this circuit to enter a state of self-sustained reverberant activity: when external stimulation is removed (time t_5_), activity within CA #2 starts to fade (again from network “periphery” towards “centre”), as the gaps appearing – and growing increasingly larger (t_6-7_) – at the rightmost ends of the raster plots on the second row show. By time t_8_, the network has returned to its initial state, with CA #5 still being “on” (self-sustained). This demonstrates that co-ctivation of CA #2 interfered only minimally with CA #5’s own activity: thanks to the strong links connecting the circuit’s kernel cells, the minor perturbation in CA #5’s halo (see orange-dashed ovals) caused by CA #2’s full ignition did not affect CA #5’s overall “on” state. Hence, the two CAs behaved as distinct, bi-stable functional units, and their superposition caused no loss of information about the identity of the co-active circuits

Using an example in which two of the five CA circuits shown in Fig. 2-Top are superposed, Fig. 3 provides a proof-of-concept demonstration that criteria (1) & (2) above are satisfied in a network trained using a Hebbian-like learning rule that closely mimics known brain mechanisms of synaptic plasticity (see detailed description in the figure’s caption); this learning rule is discussed further in the next Section.

Cell Assemblies, CAs (Braitenberg 1978; Hebb 1949; Palm 1981; von der Malsburg 1986) are sets of widely distributed, strongly and reciprocally connected cells that behave like distinct functional units having bistable character (“on” or “off” states). When deep, brain-constrained neural networks are trained using (Hebbian and “anti-Hebbian”) biologically realistic learning mechanisms (Garagnani et al. 2016, 2008; Garagnani et al. 2007; Garagnani et al. 2009a, b; Pulvermüller and Garagnani 2014; Pulvermüller et al. 2014; Schomers et al. 2017; Tomasello et al. 2018), minimally overlapping stimulus-specific assembly circuits emerge, which exhibit interesting properties. Of particular interest here, such CA circuits can “ignite” and remain active in absence of any input for long periods of time—in fact, indefinitely, if appropriate parameters are chosen (see Fig. 3A), providing a putative model correlate of working memory function (Pulvermüller and Garagnani 2014; Pulvermüller et al. 2014). Crucially, as Fig. 3B demonstrates, CA circuits can be *coactivated* without falling prey to the superposition catastrophe, by virtue of their internal structure (strong and reciprocal links between their constituent cells) and small overlap, which enable them to behave as *functionally distinct* units.

While the idea of neural activity reverberating within discrete cell assembly circuits (or synfire chains) has been around in the brain theory literature for the most part of a century (Abeles 1991; Braitenberg 1978; Hebb 1949; Mesulam 1990; Milner 1957; Palm 1981; Pulvermüller 1994; von der Malsburg 1986; Wennekers 2007), the simulation snapshots reported here (Fig. 3) are the first to document the superposition of CA circuits emerged spontaneously (i.e., via entirely unsupervised learning mechanisms) in a fully brain-constrained, multi-area neural network.

The ability of the architecture to allow two (or several, in fact) CA circuits to be co-active without interfering with each other is a consequence of the fact that such circuits share only a very small percentage of their constituent cells with each other. In other words, in the class of biologically constrained architecture considered here, the spontaneously emerging internal representations are such that their ignitions induce “quasi orthogonal” (or statistically uncorrelated) network activity states: this is because the different CA circuits happen to be almost disjoint. Empirical measures obtained with the same neural architecture show that the overlap between any two CA circuits constituted, on average, less than 5% of their component cells (see Fig. 2, bottom panel). The conditions that may enable such an emergent property of cell assemblies are discussed in the next section.

Returning to the proposal considered earlier (end of section “Superposition Catastrophe in standard neural networks”) of temporal binding via synchronous firing as a possible solution to the superposition catastrophe, if CAs do emerge in the cortex—as evidence from a growing number of experimental reports indicates (see section “Summary and Concluding Remarks” for a brief review and discussion)—it is plausible that neuronal activity might reverberate within them; if so, different frequencies, or phases of such oscillations could be used to encode different items or events, as some experimental studies appear to indicate (Canolty et al. 2010; Kerrén et al. 2022; Lundqvist et al. 2016; Vaz et al. 2020). Indeed, spontaneous oscillatory dynamics of CAs have been previously documented in a spiking brain-constrained model analogous to the present one (Garagnani et al. 2017); these results, and related computational works (Traub et al. 1996; Vicente et al. 2008), provide neuromechanistic accounts for the experimentally observed zero-lag synchronization between distant, non-directly connected cortical areas, which has been suggested to be the hallmark of widely distributed neuronal ensembles (König et al. 1995; Plenz and Thiagarajan 2007; Singer 1994). Such long-range synchronization has been reported in both humans and animals during specific cognitive tasks or in response to stimuli (Engel et al. 1991a, b; Lachaux et al. 2005; Rodriguez et al. 1999; Roelfsema et al. 1997; Supp et al. 2004; von Stein et al. 2000; see Harris and Gordon 2015 for a review).

## Why do standard DNNs fail to support cognitive superposition?

One might ask what features prevent backpropagation-trained DNNs (LeCun et al. 2015; McClelland et al. 1986; Rumelhart et al. 1986) to learn input–output mappings consisting of quasi-orthogonal vectors, which could then be coactivated without producing a “blend-like”, ambiguous output pattern. To answer this question, it is helpful to first try to understand what key characteristics brain-like networks possess which enable the emergence of stimulus-specific and mostly disjoint cell assembly circuits therein, and which are absent in standard DNNs.

A first main distinction between these two types of architectures lies in the learning mechanism used. In particular, the class of brain-constrained networks considered here (Garagnani et al. 2016, 2008; Garagnani and Pulvermüller 2011, 2016; Garagnani et al. 2009a, b; Henningsen-Schomers et al. 2023; Pulvermüller and Garagnani 2014; Schomers et al. 2017; Tomasello et al. 2017, 2018, 2019)—(see Pulvermüller et al. 2021 for a review) adopt a local synaptic plasticity rule (the “ABS rule”) which closely replicates neurophysiological phenomena known to take place in the cortex (Artola et al. 1990; Artola and Singer 1993), namely, Long-Term Potentiation (LTP)—or Hebbian synaptic strengthening—and Long-Term Depression (LTD), or ‘anti-Hebbian’ synaptic weakening (for reviews, see Bi and Poo 2001; Caporale and Dan 2008; Malenka and Bear 2004; Tsumoto 1992). The way in which these two processes of weights increase and decrease concomitantly act during the formation of cell assemblies is key to the emergence of quasi-disjoint circuits, as explained below.

In fact, by means of both Hebbian and anti-Hebbian mechanisms, a cell / node becomes “bound into” an emerging CA circuit as a result of two gradual, simultaneous processes: First, LTP induces strengthening of links between cells which are frequently coactive; hence, a node’s links to and from other cells that are—directly or indirectly—activated by the same input pattern (and which will become part of the same CA circuit) are progressively strengthened, until they reach their maximum (or “saturation”) weight value. Second, LTD leads to the weakening of connections between cells whose activities are anti-correlated; if the different input items are learned *independently* (i.e., if each of the items to be learned is presented separately), the activities of the cells that each distinct pattern activates will be anti-correlated. Thus, the links between the nodes of an emerging CA circuits and cells stimulated by other input items will be progressively weakened. This behaviour in essence implements the fundamental idea of “recruitment learning” (Valiant 2000): a node is considered recruited when it becomes *selectively* responsive to one (and only one) stimulus or item. In this sense, all cells of a given CA circuit are recruited (via Hebbian strengthening, or LTP) to respond to the same input; at the same time, they are also gradually “cut off” (through anti-Hebbian weakening, or LTD) from all other, non-relevant cells (originally linked to them), which might end up being bound into a different CA. As a result, the emerging CA circuits consist of almost disjoint sets of cells; this prevents activity within a cell-assembly circuit to be significantly affected by that of another, potentially co-active, CA circuit, thus enabling superposition.

Note that any nodes belonging to the (small) overlap between sets of cells activated by two distinct input patterns (i.e., shared by two emerging CA circuits) remain only weakly linked to the circuits’ kernels (see dashed arrows in Fig. 1B); such units are confined to the respective CA’s “halos” (Braitenberg 1978) because they consist of cells that two (or more) competing emerging CA circuits are simultaneously attempting to recruit (Garagnani et al. 2009b). It should be highlighted here that other synaptic plasticity rules—amongst which the well-known BCM rule (Bienenstock et al. 1982)—typically achieve the same temporal-competition effect between different input patterns by means of homeostatic weight scaling mechanisms, whose presence in the cortex lacks strong neuroscientific evidence (see Garagnani et al. 2009b for a discussion).

Consider now the main weight-change mechanism implemented by back-propagation, or gradient-descent learning. In a network with *n* layers, the target error-driven activity change of a node in layer *n* is reduced—namely, back-propagated—to a set of weight changes distributed across the node’s incoming links from *all* cells in layer *n*−1. These, in turn, are back-propagated to target changes in links from layer *n*−2, and so on, down to layer *n*=0, the input layer (LeCun et al. 2015; McClelland et al. 1986). This is repeated for each output node, and (many times) for each input–output pattern pair to be learned. Crucially, because of this interleaved process of “error redistribution”, *no single node of the network becomes fully selective to a specific input item*. In fact, the weights of the links in input to a node do not tend towards a bimodal distribution (with a few close to 1.0 or saturation and the rest close to 0.0, the hallmark of selectivity), but towards a *uniform* one. Hence, all cells projecting to a node remain involved—to different degrees—in determining its response to a given input. As this applies to all nodes of the hidden and output layers, the activity of *each* node of the network contributes to every successfully learned output vector. Thus, distinct input–output pairs are learned as (generally non-orthogonal) patterns of graded activities distributed over the same set (or significantly overlapping sets) of nodes, and superposition of any two of them produces a novel vector from which the original ones cannot be retrieved (refer to Fig. 1A).

A second important aspect which, in brain-constrained architectures, likely plays a role in the emergence of quasi-disjoint CA circuits is the presence of sparse and topographic between-area projections. Unlike in standard NNs, connectivity in the mammalian brain is not “all-to-all”: a single neuronal cell does not project to all cells within the adjacent cortical area (or column). Instead, synaptic projections in the cortex are typically *sparse*, patchy, and *topographic* (Amir et al. 1993; Braitenberg and Schüz 1998; Gilbert and Wiesel 1983, 1989). As a result, if two (overlapping) patterns are being superposed in area *n*, their sparse projections to area *n* + 1 will—on average—activate an overall smaller number of cells than in area *n.* Hence, the per-area number of cells that belong to the two projections’ overlap decreases from area *n* to *n* + 1 (O’Reilly and Munakata 2000, p. 291). As evidence indicates that sensory and motor information in the brain is processed by (modality preferential) hierarchies of layers, each hierarchy consisting of reciprocally and topographically linked cortical areas (e.g., Petrides and Pandya 2009; Rauschecker and Tian 2000; Ungerleider and Haxby 1994; Ungerleider and Mishkin 1982), moving further up in a processing stream is expected to lead to patterns that are progressively less overlapping. This highlights a third key aspect of brain-like architectures (in this case, shared by DNNs) which may contribute to the emergence of disjoint circuits; namely, their *deep* structure, by virtue of which patterns initially overlapping in the lowest layer of the hierarchy are gradually “pulled apart” as activity propagates towards deeper layers. This hypothesis is supported by recent neurocomputational modelling results obtained with an architecture analogous to the present one (Henningsen-Schomers et al. 2023).

Sparse between-area projections on their own, however, do not appear sufficient to guarantee the acquisition of stimulus-specific, minimally overlapping internal representations. In fact, consider, for example, a sparsely connected DNN, trained using backpropagation. Given two distinct target output patterns, the weight changes that learning each of them separately induces will end up—after a few steps of back-processing through the hidden layers—involving significantly overlapping sets of links in earlier layers (though the exact number of steps needed for this “mixing up” to happen does depend on the sparseness and topography of the connectivity). While further work investigating the use of more biologically realistic connectivity in DNNs is needed to bolster this conjecture, recent results obtained with backprop-trained networks in which the size and type of coactive input patterns—dense versus sparse—were varied (Vanegdom et al. 2022) appear to confirm the above hypothesis. It seems, therefore, that it is the presence of a local learning rule able to induce input selectivity *in conjunction* with sparse and topographic between-area projections that enables quasi-orthogonal input-specific circuits to emerge in deep neural architectures.

## Summary and concluding remarks

In this short paper I have tried to show that: (1) superposition is a basic operation that any artificial system aiming at implementing human-like, general intelligence should support; (2) deep, brain-constrained architectures with biologically realistic learning and connectivity exhibit the emergence of internal circuits which, by virtue of their structural properties—i.e., minimal overlap—and dynamics, provide a natural substrate for the implementation of superposition; and (3) backpropagation training of standard DNNs leads to internal representations that are generally non-orthogonal (i.e., patterns of graded activities uniformly distributed over the same hidden nodes), hence inadequate to support this function.

The claim emerging from the above is that, in order to explain a key cognitive ability the human brain effortlessly supports, something more “brain-constrained” than DNNs with all-to-all connectivity and gradient-descent training is needed (Pulvermüller 2023; Pulvermüller et al. 2021). Deep network architectures with Hebbian and anti-Hebbian learning mechanisms which spontaneously develop quasi-orthogonal, input-selective, functionally distinct and distributed CA circuits may be a possible answer. This claim, however, does not rule out the possibility that these two types of neural codes (graded and mostly overlapping vs. discrete and quasi-orthogonal—see Fig. 1A, B, respectively) may coexist in the cortex, and be used as and when appropriate, depending on the specific task at hand. In fact, using non-orthogonal graded-activity vectors can be advantageous when a system’s response should change in a “smooth-like”, continuous manner as a function of gradual changes in its input. This behaviour may underlie, for example, part of our ability to generalise across perceptually similar items (O’Reilly and Munakata 2000). Due to their discrete, bistable (“on” or “off”) character, CA circuits do not exhibit such flexible behaviour: presenting an input pattern that differs from the learned, “familiar” one a CA circuit is selective to can produce either the circuit’s full ignition, or the absence thereof, but nothing in between. Thus, graded differences in the input are *discretised* into “all-or-none” responses, which would seem to make a CA-based architecture sub-optimal when it comes to implementing a generalisation mechanism based on degrees of similarity. On the other hand, CA circuits offer a higher level of robustness than codes relying solely on fully distributed patterns. In fact, a neocortical CA circuit is estimated to include from a few thousands to several tens of thousands neurons (Palm 1993). Notably, all cells of a CA circuit are recruited to perform the same function—namely, to become active (or switch “on”) only in presence of a specific input pattern and remain inactive (“off”) otherwise; such a high degree of redundancy makes CA circuits extremely fault tolerant and resilient to noise. The same cannot be said of fully distributed architectures, characterised by states of graded activity in which, as argued in the previous section, the specific activity (or lesion) of a single node may have a significant impact on the overall network’s output.

One question that still awaits a conclusive experimental answer is whether quasi-orthogonal, input-selective, distributed CA circuits like those observed in the present simulations actually emerge in the cortex. A growing body of evidence providing indirect support for the presence of cell assembly-like activity in the brain comes from neuroimaging studies in humans, single-cell recordings in animals, as well as invasive recordings in patients. For example, a number of studies have identified patterns of synchronized neural activity in response to stimuli or during specific cognitive tasks, which have been interpreted as reflecting activity reverberating within specific cell assembly circuits (Buzsáki 2004; Canolty et al. 2010; Gray et al. 1989; Kreiter and Singer 1992, 1996; Pulvermüller et al. 1995). Others have documented larger neurophysiological (including oscillatory) responses to familiar, meaningful stimuli than to unknown, senseless material, taking this to index the ignition of corresponding learnt CA circuits—and the absence thereof, respectively—in the cortex (Canolty et al. 2007; Craddock et al. 2015; Gao et al. 2013; Garagnani et al. 2009a, b; Hassler et al. 2011; Krause et al. 1998; Lutzenberger et al. 1994; Mainy et al. 2008; Pulvermüller et al. 1996, 2001; Shtyrov and Pulvermüller 2002; Tallon-Baudry et al. 1996). Direct experimental evidence for the existence of CA circuits in the cortex, however, remains elusive (for a review and perspective, see Buzsáki 2010). Given this, demonstrating that such putative circuits are mostly disjoint (i.e., that the activity states they induce are quasi orthogonal) may seem an even harder enterprise. That said, sparse and approximately orthogonal neural activity has been actually documented in the so-called “place cells” of the rodent hippocampus during spatial navigation tasks (Barnes et al. 1990; O’Keefe and Dostrovsky 1971; Pfeiffer and Foster 2013; Wilson and McNaughton 1993). Intriguingly, some recent studies did report the presence of an orthogonal neural code also in the neocortex (Flesch et al. 2022; Gennari et al. 2021; Mao et al. 2017). Additionally, one main strategy the cortex may use to achieve orthogonality between its representations consists of adopting a *sparse* (and distributed) code. In fact, if a CA circuit comprises a very small fraction of the full set of cortical neurons, given a random and patchy distribution of CA cells over the two hemispheres, the probability of any two circuits to exhibit a substantial overlap is very low; furthermore, as a result of the recruitment learning mechanisms, such overlap is expected to be relegated to the “halo” part of the assemblies (see Fig. 1B). In practice, this leads to mostly disjoint circuits. Indeed, there is substantial evidence indicating that the cortex does make use of sparse representations (e.g., R. Baddeley et al. 1997; Beyeler et al. 2019; Cox and Riesenhuber 2015; Jääskeläinen et al. 2022; Liang et al. 2019; Olshausen and Field 1996; Reddy and Kanwisher 2006; Rolls and Tovee 1995; Tang et al. 2018; Vinje and Gallant 2000). Therefore, while direct, conclusive proof for the existence of quasi-orthogonal cell assembly circuits in the cortex is still missing, a large body of independent results (ranging from single-cell recordings and neuroimaging studies—see above—to neuroanatomical data about sparse connectivity, to solid evidence for the existence of neurophysiological processes implementing both Hebbian—LTP—and anti-Hebbian—LTD—learning) together provide compelling evidence in support of this hypothesis.

In line with the above, some scholars have started to explore the use of more biologically accurate, sparse connectivity and Hebbian mechanisms in (deep) feedforward and recurrent NNs (Amit 2019; Bahroun et al. 2017; Bolcskei et al. 2019; Frenkel et al. 2021), suggesting this may be a fruitful future direction in the emerging area of cognitive AI systems.

Investigating how the type of between-area connectivity affects the superposition and working memory capacities of deep brain-like networks also seems an important future avenue of research. In fact, previous simulations carried out with a (spiking) model similar to the present one showed that the between-area (so-called “jumping”) links connecting non-adjacent brain regions (present in humans but absent or weaker in nonhuman primates) lead to superior verbal WM skills, providing a possible explanation for our species-unique language abilities (Schomers et al. 2017). Following up on this work, in novel experiments carried out with a brain-like architecture analogous to the present one we investigated the properties of networks having different “depths”, and, hence, developing cell assembly circuits with different total-area spans (Garagnani et al., *in preparation*). Preliminary results show that, as the hierarchy depth increases, so does the *maximal* number of CA circuits the system is able to superpose. Intriguingly, such a ‘superposition capacity’ appears to asymptote as hierarchical depth increases. This would suggest the existence of an architectural upper bound on the maximum number of CA circuits that may be coactive, which could be directly related to the well-known limited capacity of human working memory (Cowan 2001; Cowan et al. 2007). While these predictions await statistical (and experimental) validation, they point to a possible factor that could help explain the phylogenetic growth in size of the human brain, unmatched by that of our closest nonhuman primate relatives (Avants et al. 2006; Preuss 2011). In particular, such preliminary computational results suggest that the significant expansion of cortical-association areas in humans (leading to an increase in the network’s depth) could have been driven, in part, by the  resulting evolutionary advantage provided by better working-memory skills. The ability to maintain several items simultaneously active in WM—while preserving their original features—appears crucial, amongst other things, for enabling the emergence of theory-of-mind and social-cognition skills (Meyer and Collier 2020; Noguchi et al. 2022), and the construction of a “language-ready” brain (Arbib 2009, 2017).

To conclude, it is remarkable that, although the inherently fully-distributed and graded character of the representations learned by backpropagation-trained nets may well be inadequate to support superposition, the multi-layer structure of D/CNNs—a pervasive feature of information processing in the cortex—might turn out to be a pre-requisite for the emergence of some of the fundamental building blocks of cognition. Just like the implementation of neurobiologically accurate computational models, historically motivated by questions concerning brain function, is now a promising direction for the development of new cognitive AI systems, the use of deep architectures, standard practice in nowadays artificial NNs, may be key in future large-scale brain-constrained modelling efforts to gaining a better understanding of human intelligence.

## Data Availability

All datasets generated during and/or analysed for the current study are available upon request from the author.
